# Genetic SHBG Deficiency Unmasks the Discrepancy Between Biochemical and Clinical Hypogonadism

**DOI:** 10.1210/jcemcr/luaf273

**Published:** 2025-11-21

**Authors:** Xue D Manz, Michel J Vos, Ron S Booij, Rieneke Sanson, Leo H J Jacobs

**Affiliations:** Laboratory for Clinical Chemistry and Hematology, Meander Medical Centre, 3813 TZ Amersfoort, The Netherlands; Department of Laboratory Medicine, University of Groningen, University Medical Center Groningen, 9700 RB Groningen, The Netherlands; Department of Laboratory Medicine, University of Groningen, University Medical Center Groningen, 9700 RB Groningen, The Netherlands; Department of Internal Medicine, Meander Medical Centre, 3813 TZ Amersfoort, The Netherlands; Laboratory for Clinical Chemistry and Hematology, Meander Medical Centre, 3813 TZ Amersfoort, The Netherlands

**Keywords:** SHBG, testosterone, hypogonadism, genetic variants

## Abstract

Near–absent serum SHBG influences calculated free testosterone, complicating the evaluation of suspected hypogonadism. We report a 50–year–old man with longstanding low total testosterone (147.1 ng/dL [SI: 5.1 nmol/L], reference range: 289-866 ng/dL [SI: 10.0-30.0 nmol/L]). Further laboratory testing revealed a suppressed SHBG of 2 nmol/L (reference range: 14–72 nmol/L) and remarkably normal calculated free testosterone (between 5.0 and 6.0 ng/dL [SI: 0.174-0.210 nmol/L], reference range: 4.9-15.7 ng/dL [SI: 0.160-0.700 nmol/L]). Direct equilibrium–dialysis measurement, however, revealed low free testosterone (2.9 ng/dL [SI: 0.099 nmol/L], reference range: 4.9-15.8 ng/dL [SI: 0.170-0.546 nmol/L]), establishing the diagnosis of biochemical hypogonadism. Genetic analysis demonstrated compound heterozygosity for the *SHBG* variants c.554C > T (p.Pro185Leu), which impairs steroid binding and increases clearance, and c.670G > A (p.Gly224Arg), which prevents secretion. This double hit explains the paradox of marked biochemical abnormalities in the absence of clinical hypogonadism. The case underscores not only the limitations of calculated, and even directly measured, free testosterone when SHBG is profoundly low but also the importance of integrating genetic analysis and clinical context into the diagnostic process. This case suggests that in rare cases of genetic SHBG deficiency, male reproductive function may remain intact despite profoundly low circulating SHBG levels. Clinicians should recognize that SHBG genetic variants may cause aberrant biochemical findings that mimic hypogonadism, which should be included in the differential diagnosis when evaluating patients with unexplained hormonal abnormalities.

## Introduction

SHBG is a glycoprotein primarily produced in the liver and is critical for the transport of sex steroids. SHBG has a high affinity for androgens and a lower affinity for estrogens [1]. SHBG regulates the free, biologically active fractions and influences both their metabolic clearance and tissue accessibility [2]. Modulating the proportion of unbound sex steroids orchestrates sexual characteristics and reproductive function in both men and women. Reduced SHBG levels may have significant clinical consequences and are commonly associated with obesity, insulin resistance, metabolic syndrome, and polycystic ovary syndrome. SHBG levels are regulated by multiple factors, including hormonal, nutritional, genetic, and metabolic influences. Circulating SHBG concentrations are shaped by hormonal, nutritional, metabolic, and genetic factors. Twin and family studies indicate that common genetic variants explain approximately 30% to 80% of the interindividual variation in serum SHBG levels [3]. By contrast, true monogenic SHBG deficiency due to loss-of-function mutations is exceedingly rare. Although numerous SHBG polymorphisms have been identified, inconsistencies among published studies continue to obscure their precise biological significance. In this report, we present an adult man with near-absent SHBG levels, caused by a compound heterozygous variation in the coding region of human *SHBG*, illustrating diagnostic pitfalls and sex-specific phenotypic effects.

## Case Presentation

A 50-year-old man was referred to the internal medicine department for reevaluation of a longstanding low testosterone level. His condition was first discovered 10 years earlier during a fertility assessment, when semen analysis revealed oligoasthenoteratozoospermia. At that time, laboratory evaluation showed a total testosterone of 147.1 ng/dL (SI: 5.1 nmol/L) (reference range: 289-866 ng/dL [SI: 10.0-30.0 nmol/L]), reduced inhibin B of 86 pg/mL (SI: 86 ng/L) (reference range: 150-400 pg/mL [SI: 150-400 ng/L]), and discordant gonadotropins: an inappropriately low LH of 1.8 IU/L (reference range: 1.5-8 IU/L) alongside an elevated FSH of 13.6 IU/L (reference range: 2-7 IU/L).

Therapeutic intervention included clomiphene citrate therapy and left-sided varicocelectomy, which improved spermatogenesis. His partner initially conceived spontaneously but experienced several miscarriages. Eventually, the couple had 2 children while he continued clomiphene therapy for several years. Notably, no further investigation into the underlying cause of his hypogonadism was performed at the fertility center.

Upon presentation to our hospital, the patient appeared generally healthy with normal libido, energy levels, and muscle strength, which was incongruent with his biochemical profile. Physical examination revealed a well-virilized male with normal male-pattern hair distribution, low-normal testicular volumes (12 mL, reference range: 12-25 mL), normal semen volume (3.3 mL, reference range: 1.5-5 mL), and no gynecomastia.

## Diagnostic Assessment

Initial laboratory evaluation during clomiphene therapy revealed some striking findings (Table 1). A low total testosterone of 115 ng/dL (SI: 4.0 nmol/L) (reference range: 230-864 ng/dL [SI:10.0-30.0 nmol/L]) with a LH of 7 IU/mL (reference range: 1-12 IU/mL) and suppressed SHBG of 2 nmol/L (reference range: 14-72 nmol/L). IGF-1 was also reduced at 48 ng/mL (−2.5 SD score). Furthermore, thyroid function (TSH and free T4) and adrenal function (morning cortisol) were normal (Table 1). Brain magnetic resonance imaging showed no pituitary abnormalities.

**Table 1.** Laboratory test results

Laboratory test	Results at presentation (on clomiphene)	6 weeks past presentation	12 weeks past presentation	Reference range
TSH	0.64 mIU/L			0.35-5.00 mIU/L
FT4	0.86 ng/dL (11.2 pmol/L)			0.7-1.5 ng/dL (9-19 pmol/L)
Cortisol 10:00 AM	14.1 µg/dL (0.39 µmol/L)			3.6-19.6 µg/dL (0.10-0.54 µmol/L)
LH	7 IU/L	2 IU/L	4 IU/L	1-12 IU/L
Testosterone	115 ng/dL (4.0 nmol/L)	141 ng/dL (4.9 nmol/L)	104 ng/dL (3.6 nmol/L)	230-864 ng/dL (8.0-30 nmol/L)
Testosterone (LC-MS/MS)	85 ng/dL (2.95 nmol/L)			302-1066 ng/dL (10.5-37 nmol/L)
Calculated free Testosterone	5.0 ng/dL (0.175 nmol/L)	6.0 ng/dL (0.210 nmol/L)	8.5 ng/dL (0.241 nmol/L)	4.6-20.2 ng/dL (0.160-0.700 nmol/L)
Free Testosterone (dialysis, LC-MS/MS)	2.9 ng/dL (0.0987 nmol/L)			4.9-15.8 ng/dL (0.170-0.546 nmol/L)
SHBG	2 nmol/L	2 nmol/L	1 nmol/L	14-72 nmol/L
IGF-1 (SDS)	48 ng/mL (−2.5 SDS)	153 ng/mL (−0.06 SDS)		

The patient was taking clomiphene at presentation, which was discontinued after the visit.

Values in parentheses are International System of Units.

Abbreviations: FT4, free thyroxine 4; LC-MS/MS, liquid chromatography-tandem mass spectrometry; LH, luteinizing hormone; SDS, SD score; SHBG, sex hormone binding globulin; TSH, thyroid stimulating hormone.

Although his free testosterone of 5.0 ng/dL (SI: 0.174 nmol/L) (reference range: 4.9-15.7 ng/dL [SI: 0.160-0.700 nmol/L]) was normal, it was calculated based on SHBG levels [2]. Therefore, direct free testosterone was assessed by equilibrium dialysis through our reference laboratory, which revealed free testosterone of 2.9 ng/dL (SI: 0.099 nmol/L) (reference range: 4.9-15.8 ng/dL [SI: 0.170-0.546 nmol/L]), together with a reduced total testosterone of 85 ng/dL (SI: 2.95 nmol/L) (reference range: 302-1065 ng/dL [SI: 10.5-37 nmol/L]), determined by liquid chromatography-tandem mass spectrometry. Although direct measurement revealed low free testosterone, the absence of clinical symptoms suggested preserved androgen action at the tissue level. This discrepancy prompted further investigation.

Given the persistently near-absent SHBG level without clinical manifestation, genetic evaluation of *SHBG* was performed. Informed consent was obtained from the patient for genetic analysis and publication. Genomic DNA was isolated from peripheral blood leukocytes. The genomic sequence for human *SHBG* was retrieved from the National Center for Biotechnology Information database (Gene ID 6462). The *SHBG* gene was amplified from genomic DNA in 3 overlapping fragments, using DreamTaq DNA polymerase (Thermo Fisher Scientific, Groningen, The Netherlands). Sequencing was performed by Sanger sequencing (BaseClear, Leiden, The Netherlands) and analyzed using BioEdit 7.7.1 [4]. Sequencing identified 2 heterozygous single nucleotide polymorphisms (SNPs) in the coding region of *SHBG.* For verification of the 2 SNPs, restriction enzyme analysis was carried out on the PCR products using the restriction enzymes SmaI and Cfr10 I (New England Biolabs, Ipswich, USA and NIPPON Genetics, Düren, Germany), respectively. The presence of the SNPs resulted in disruption of the enzyme recognition sites.

## Treatment

Clomiphene stimulates pituitary gonadotropins by modulating the estrogen receptor. This binding results in LH and FSH release and negatively regulates GH production by activating SOCS3, which inhibits Jak/Stat signaling. Subsequently, this reduces IGF-1 levels by 40% to 80% in healthy adult men with intact pituitary function [5]. Therefore, the patient's clomiphene therapy was discontinued to evaluate his normal hormonal regulation. Furthermore, no testosterone supplementation was initiated because the patient did not experience any clinical symptoms.

## Outcome and Follow-up

Six weeks after clomiphene had been discontinued, the patient's IGF-1 concentration had normalized to 153 ng/mL (−0.06 SD score), whereas SHBG remained markedly suppressed (<5 nmol/L). Consequently, after respectively 6 and 12 weeks, total testosterone continued to fluctuate between 104 and 141 ng/dL (SI: 3.6-4.9 nmol/L), yet the calculated free testosterone stayed within the reference range at 6.95 ng/dL (SI: 0.241 nmol/L). Despite the persistently low total testosterone, the patient remained clinically asymptomatic.

**Figure 1.**
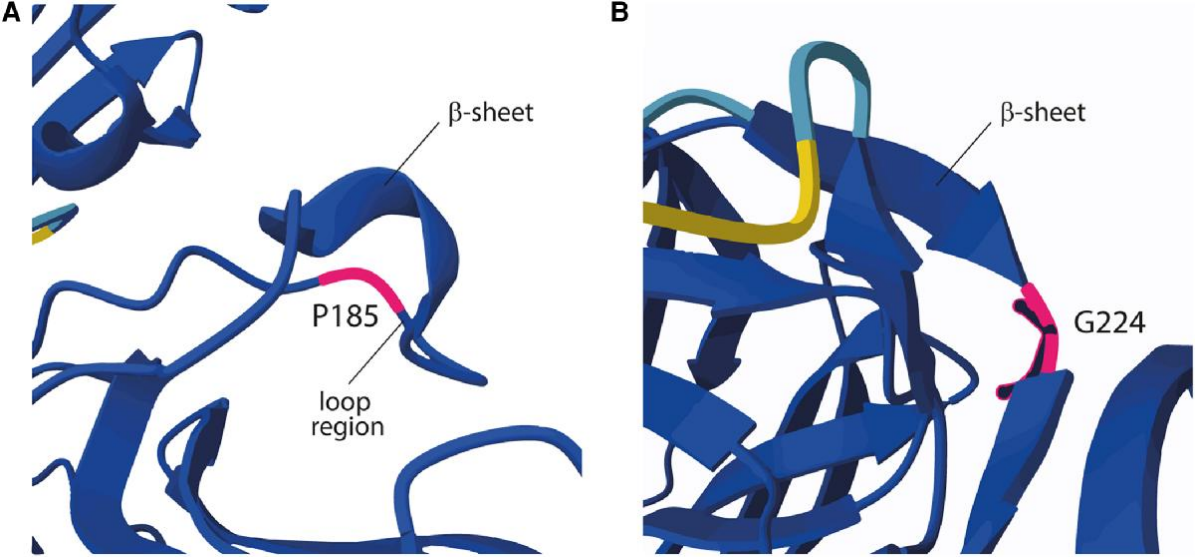
AlphaFold-predicted structure of SHBG illustrating the location and predicted effects of the identified variants. (A) P185 is situated within a loop region adjacent to a β-sheet. Proline's rigid cyclic structure restricts flexibility. The p.P185L substitution replaces proline with leucine, a more flexible residue, which may alter loop dynamics and protein stability. (B) G224 is positioned between 2 β-sheets, a crucial structural feature required for local flexibility and β-sheet alignment. The p.G224R substitution introduces a bulkier, positively charged arginine, which could disrupt local folding through steric clashes and altered electrostatic interactions. Abbreviations: G224, glycine 224; P185, proline 185.

The 2 SNPs correspond to the National Center for Biotechnology Information reference SNP cluster IDs rs6258 and rs1182086432, resulting in a proline to leucine (exon 4, c.554C > T, p.P185L) amino acid substitution and a glycine to arginine (exon 5, c.670G > A, p.G224R) amino acid substitution, respectively. 3D structural modeling using AlphaFold [6] predicted that Pro185 is located in a loop region, where it likely confers rigidity (Fig. 1A). Its substitution with Leu185 introduces greater flexibility, potentially impacting protein folding or function. AlphaFold modeling of Gly224 showed that this amino acid is positioned between 2 β-sheets, where it likely facilitates flexibility and structural integrity. Substitution to Arg224 introduces a positively charged, bulkier side chain, which is predicted to cause reduced flexibility, steric hindrance, and most likely structural disruption (Fig. 1B).

## Discussion

This case highlights the complex interplay between biochemical evaluation and clinical presentation in endocrinology. SHBG plays an important role in testosterone homeostasis, and severely low levels can result in misleading calculated free testosterone values. Free testosterone is estimated by mathematically modeling its equilibrium with SHBG and albumin, using measured total testosterone, SHBG, and albumin levels, according to the Vermeulen calculation [7]. When SHBG is profoundly low, calculated free testosterone may not reflect dialyzed free testosterone, and in such discordant situations, equilibrium dialysis provides a more definitive assessment [8].

The patient’s markedly reduced SHBG levels were explained by a compound heterozygous missense variant in the *SHBG* gene: c.554C > T (p.Pro185Leu; rs6258) in exon 4 and c.670G > A (p.Gly224Arg; rs182086432) in exon 5. These *SHBG* variants have been previously characterized. The p.Pro185Leu variant is suggested to have a prevalence of 2% and reduces SHBG levels by 25% to 50% in heterozygous subjects [9]. The substitution of proline by leucine at position 185 may lead to a more flexible structure, affecting glycosylation and calcium binding, ultimately reducing testosterone binding affinity and increasing SHBG clearance (Fig. 1A). This altered binding affinity is supported by a recent study by Walravens et al [10]. Interestingly, this study also showed that the rs6258 variant did not affect calculated free testosterone values. This is probably explained by the fact that less than 20% of circulating SHBG has reduced testosterone affinity, while the remaining 80% retains normal function. However, the participants were healthy men carrying a heterozygous SNP. A case report with a homozygous mutation showed a SHBG serum level below 5 nmol/L and described gonadotropin-independent precocious puberty, likely due to increased free sex hormone levels [11]. Furthermore, the patient we describe is compound heterozygous for 2 SNPs, in which the rs182086432, p.Gly224Arg variant has been identified in a Dutch family and is associated with intracellular retention and a complete block in SHBG secretion [12]. Substitution to Arg224 is likely to impair SHBG folding, which causes accumulation of SHBG in the endoplasmic reticulum, preventing secretion [12]. As a result, homozygous variants have no detectable circulating SHBG. Interestingly, the homozygous presence in a male showed normal volume and sperm concentration in his semen analysis. The female exhibited a prolonged but regular menstrual cycle without signs of hirsutism or hyperandrogenism. This suggests that a complete absence of circulating SHBG does not interfere with gonadal function [2].

Total to nearly absent SHBG levels did not interfere with male gonadal differentiation or pubertal development and reproductive function of the gonads. In contrast, in women, low SHBG increases bioavailable androgens, potentially causing hirsutism, menstrual irregularities, and features of polycystic ovary syndrome. This sex-specific difference may be explained by various mechanisms. First, SHBG is also produced in other tissues than the liver, including the placenta, testis, brain, and endometrium. The testis produces an alternative SHBG transcript [3], also known as androgen binding protein. This transcript lacks exon 7, which may result in different protein folding to provide local regulatory functions [13]. The mutations found in this patient are located in exon 4 and 5, which are identical for hepatic and testicular SHBG. Therefore, we expect that testicular SHBG (androgen binding protein) is also affected, although this hypothesis is an area for future study. Second, intratesticular testosterone concentrations are approximately 100-fold higher than serum levels, which exceeds testicular SHBG binding capacity, probably explaining the preservation of spermatogenesis in this patient [14, 15]. The coexisting andrological abnormalities (eg, low inhibin B/high FSH, oligoasthenoteratozoospermia, varicocele, small testicular volume) can explain gonadotropin and semen findings but do not account for the near-absence of SHBG caused by *SHBG* variants in this patient as circulating SHBG is from hepatic origin [3, 13].

In summary, the compound heterozygosity of SHBG variants identified in our patients describes preserved androgen-dependent functions and normal LH that argue against clinical hypogonadism. This highlights the limitations of biochemical criteria and underscores the need to contextualize laboratory values with clinical presentation, particularly in the setting of genetic SHBG deficiencies. In such rare cases, the concept of “biochemical hypogonadism” may not indicate true androgen insufficiency but rather reflect limitations in current diagnostic paradigms. Long-term follow-up will be important to determine whether such patients face any late complications from their lifelong absence of circulating SHBG.

## Learning Points

- Free testosterone must be measured directly by equilibrium dialysis when SHBG levels are extremely low, since calculated values are invalid.
- Compound heterozygosity for p.Pro185Leu (affecting protein stability/binding) and p.Gly224Arg (causing retention) can reduce circulating SHBG.
- Male gonadal function and fertility can remain largely intact despite absent circulating androgen carrier protein; most likely, intratesticular testosterone concentrations are independent of plasma binding proteins.

## Contributors

All authors contributed to the diagnosis and clinical interpretation of the patient's case. X.D.M. conceptualized and prepared the manuscript. L.H.J. supervised the project. M.J.V. and R.S.B. performed and interpreted the DNA sequencing analysis. R.S. was responsible for patient care. All authors reviewed and approved the final version.

## Data Availability

Some or all datasets generated during and/or analyzed during the current study are not publicly available but are available from the corresponding author on reasonable request.
